# Supplementary figures and images for: Knowledge, behaviours and attitudes towards Evidence-Based Practice amongst physiotherapists in Poland. A nationwide cross-sectional survey and focus group study protocol
Source: PLoS One. 2022 Mar 1;17(3):e0264531. doi: 10.1371/journal.pone.0264531 (PMC8887773; doi:10.1371/journal.pone.0264531)

STUDY 1: Quantitative Study

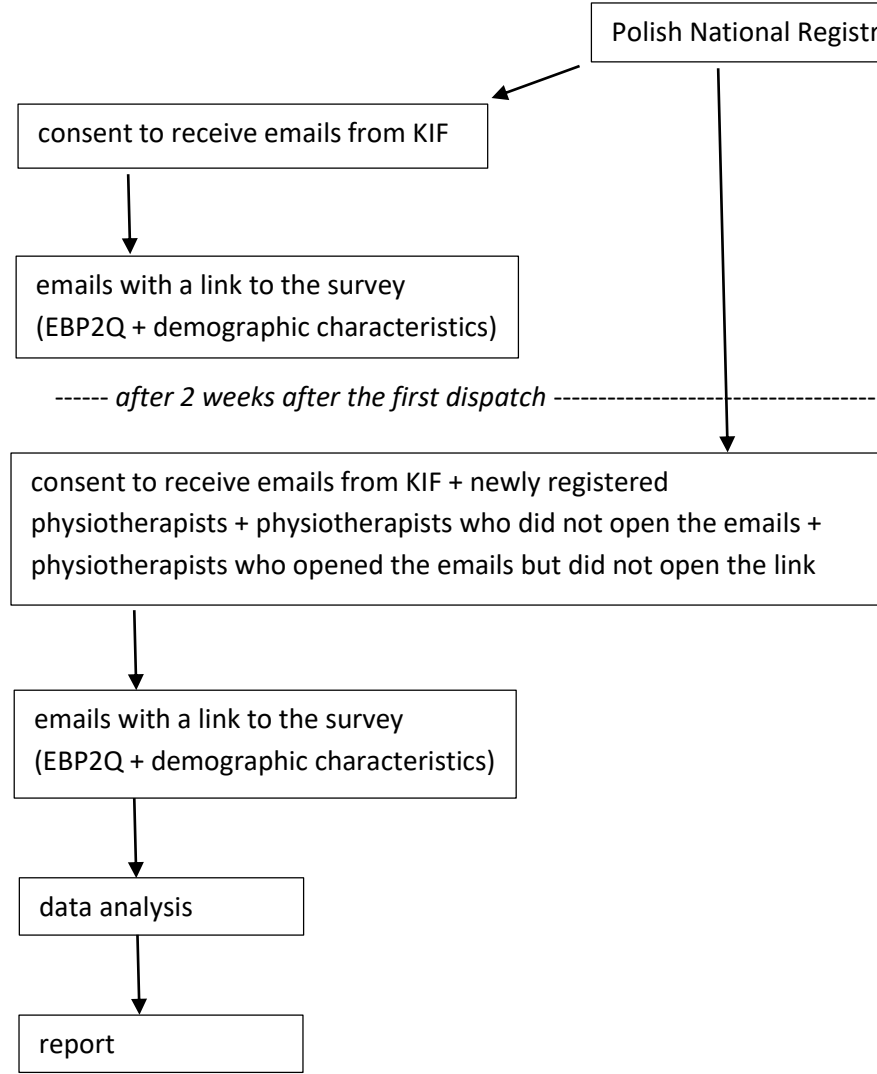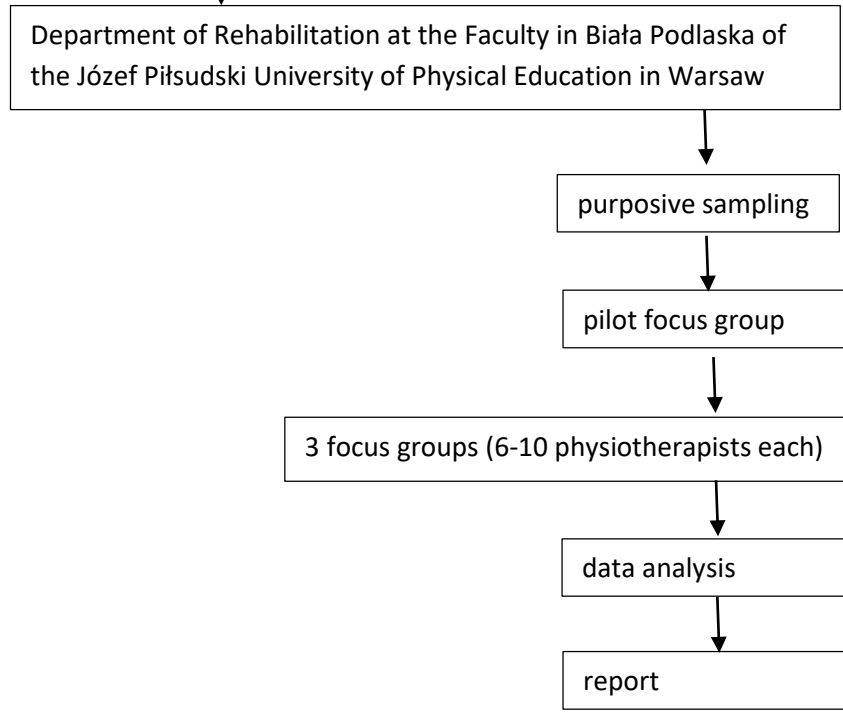

STUDY 2: Qualitative Study

Supplement: S1 File — (PDF) [file pone.0264531.s001.pdf]
