# Supplementary material for: Knowledge, behaviours and attitudes towards Evidence-Based Practice amongst physiotherapists in Poland. A nationwide cross-sectional survey and focus group study protocol
Source: PLoS One. 2022 Mar 1;17(3):e0264531. doi: 10.1371/journal.pone.0264531 (PMC8887773; doi:10.1371/journal.pone.0264531)
Supplement: S2 File — (PDF) [file pone.0264531.s002.pdf]

Wiedza, zachowania i postawy fizjoterapeutów wobec praktyki opartej na danych naukowych (EBP2Q)

0% — 100%

Szanowni Państwo,

zwracamy się z prośbą o udział w badaniu ankietowym online, dotyczącym praktyki opartej na danych naukowych (*Evidence-Based Practice, EBP*). Jest to badanie z wykorzystaniem kwestionariusza *Evidence-Based Practice Profile (EBP2) Questionnaire* w polskiej wersji językowej. Celem badania jest poznanie wiedzy, zachowań i postaw polskich fizjoterapeutów wobec EBP.

Badanie jest jednym z elementów projektu naukowego "PRO-EBP Platforma promowania i upowszechniania praktyki zawodowej opartej na wiedzy naukowej", realizowanego w ramach programu "Społeczna odpowiedzialność nauki" Ministra Edukacji i Nauki, SONP/SP/461408/2020. Projekt jest prowadzony w Akademii Wychowania Fizycznego Józefa Piłsudskiego w Warszawie, we współpracy z Krajową Izbą Fizjoterapeutów i PEDro Partnership, Uniwersytet w Sydney.

Badanie jest dobrowolne i anonimowe, a zebrane dane (odpowiedzi na pytania) będą wykorzystane wyłącznie dla celów naukowych. Wszelkie informacje mogące zidentyfikować osobę wypełniającą ankietę pozostaną chronione.

Instrukcja

Wypełnienie ankiety zajmuje około 10-12 minut.

Proszę zaznaczyć jedną odpowiedź w każdym wierszu.

Można także podać swój komentarz zamieszczając go w polu pod każdą tabelą z pytaniami.

Będziemy bardzo wdzięczni za poświęcenie czasu i uwagi na wypełnienie ankiety.

Zespół badawczy

Dalej

Stwórz własną ankietę na Webankieta.pl

Wiedza, zachowania i postawy fizjoterapeutów wobec praktyki opartej na danych naukowych (EBP2Q)

4% — 100%

Proszę ocenić trafność poszczególnych stwierdzeń w skali 1-5: *

Można udzielić jednej odpowiedzi w wierszu.

	zdecydowanie się nie zgadzam (1)	nie zgadzam się (2)	nie mam zdania (3)	zgadzam się (4)	zdecydowanie się zgadzam (5)
1. Znam znaczenie terminu Evidence – based Practice (EBP)	○	○	○	○	○
2. Mam świadomość istnienia EBP w moim zawodzie	○	○	○	○	○
3. EBP stanowi nowoczesny model wykonywania mojego zawodu	○	○	○	○	○
4. Mam świadomość ciągłego rozwoju EBP w moim zawodzie	○	○	○	○	○

Czy ma Pani / Pan dodatkowe komentarze dotyczące udzielonych odpowiedzi?

Wpisz...

Wstecz Dalej

Stwórz własną ankietę na Webankieta.pl

Wiedza, zachowania i postawy fizjoterapeutów wobec praktyki opartej na danych naukowych (EBP2Q)

13% — 100%

Proszę ocenić trafność poszczególnych stwierdzeń w skali 1-5: *

Można udzielić jednej odpowiedzi w wierszu.

	z pewnością nie (1)	mało prawdopodobne (2)	rozważę taką możliwość (3)	bardzo prawdopodobne (4)	z pewnością tak (5)
5. Zamierzam poszerzać własną wiedzę na temat EBP	○	○	○	○	○
6. Zamierzam rozwijać własne umiejętności w zakresie dostępu i oceny dowodów naukowych istotnych dla mojej praktyki zawodowej	○	○	○	○	○
7. Zamierzam korzystać z odpowiedniej literatury naukowej w celu aktualizacji posiadanej wiedzy	○	○	○	○	○
8. Zamierzam zastosować najlepsze dostępne dowody naukowe w celu poprawy jakości własnej praktyki zawodowej	○	○	○	○	○

Czy ma Pani / Pan dodatkowe komentarze dotyczące udzielonych odpowiedzi?

Wpisz...

Wstecz Dalej

Stwórz własną ankietę na Webankieta.pl

Wiedza, zachowania i postawy fizjoterapeutów wobec praktyki opartej na danych naukowych (EBP2Q)

21% — 100%

Proszę ocenić trafność poszczególnych stwierdzeń w skali 1-5: *

Można udzielić jednej odpowiedzi w wierszu.

	zdecydowanie się nie zgadzam (1)	nie zgadzam się (2)	nie mam zdania (3)	zgadzam się (4)	zdecydowanie się zgadzam (5)
9. Zastosowanie EBP w mojej praktyce zawodowej jest niezbędne	○	○	○	○	○
10. Doniesienia naukowe są przydatne w mojej codziennej praktyce zawodowej	○	○	○	○	○
11. Powinnam / powinienem częściej wykorzystywać dowody naukowe w mojej codziennej praktyce zawodowej	○	○	○	○	○
12. Jestem zainteresowana / zainteresowany poprawą umiejętności niezbędnych do włączenia EBP do mojej praktyki zawodowej	○	○	○	○	○
13. Zastosowanie EBP poprawia jakość mojej praktyki zawodowej	○	○	○	○	○
14. Zastosowanie EBP pomaga mi podejmować właściwe decyzje kliniczne dotyczące pacjentów podczas mojej praktyki zawodowej	○	○	○	○	○
15. W mojej codziennej praktyce zawodowej niektóre wymogi EBP mogą mieć ograniczone zastosowanie ze względu np. na brak sprzętu, procedur, personelu, itd.	○	○	○	○	○
16. Zastosowanie EBP w mojej codziennej praktyce zawodowej nie ma sensu z powodu braku dowodów naukowych na poparcie skuteczności większości wykonywanych przeze mnie czynności zawodowych	○	○	○	○	○
17. Zastosowanie EBP nie uwzględnia preferencji moich pacjentów	○	○	○	○	○
18. W podejmowaniu właściwych decyzji w mojej praktyce zawodowej wyżej cenię doświadczenie kliniczne niż wyniki badań naukowych	○	○	○	○	○
19. Doświadczenie kliniczne jest najlepszym sposobem oceny skuteczności danego działania	○	○	○	○	○
20. Krytyczny przegląd piśmiennictwa naukowego i jego znaczenie dla skuteczności leczenia/opieki nad pacjentami nie ma zastosowania w praktyce zawodowej	○	○	○	○	○
21. Poszukiwanie istotnych dowodów naukowych nie ma zastosowania w praktyce zawodowej	○	○	○	○	○

Czy ma Pani / Pan dodatkowe komentarze dotyczące udzielonych odpowiedzi?

Wpisz...

Wstecz | Dalej

Stwórz własną ankietę na Webankieta.pl

Wiedza, zachowania i postawy fizjoterapeutów wobec praktyki opartej na danych naukowych (EBP2Q)

29% — 100%

Proszę ocenić stopień zrozumienia poszczególnych pojęć: *

Można udzielić jednej odpowiedzi w wierszu.

	nigdy o nim nie słyszałam/em (1)	słyszałam/em o nim, jednak nie znam jego znaczenia (2)	częściowo rozumiem (3)	całkiem dobrze rozumiem (4)	rozumiem i potrafię wytłumaczyć innym jego znaczenie (5)
22. Ryzyko względne (RR – Relative risk)	○	○	○	○	○
23. Ryzyko bezwzględne (AR – Absolute risk)	○	○	○	○	○
24. Przegląd systematyczny (Systematic review)	○	○	○	○	○
25. Iloraz szans (OR – Odds ratio)	○	○	○	○	○
26. Metaanaliza (Meta-analysis)	○	○	○	○	○
27. NNT - number needed to treat	○	○	○	○	○
28. Przedział ufności (CI – Confidence interval)	○	○	○	○	○
29. Złudzenie publikacyjne (Publication bias)	○	○	○	○	○
30. Wykres leśny (Forest plot)	○	○	○	○	○
31. Zamiar leczenia (Intention to treat)	○	○	○	○	○
32. Istotność statystyczna (Statistical significance)	○	○	○	○	○
33. Minimum clinically worthwhile effect	○	○	○	○	○
34. Znaczenie kliniczne (Clinical importance)	○	○	○	○	○
35. Randomizowane badanie kliniczne (RCT – Randomised controlled trial)	○	○	○	○	○
36. Wyniki dychotomiczne (Dichotomous outcomes)	○	○	○	○	○
37. Wyniki ciągłe (Continuous outcomes)	○	○	○	○	○
38. Wielkość efektu leczenia (Treatment effect size)	○	○	○	○	○

Czy ma Pani / Pan dodatkowe komentarze dotyczące udzielonych odpowiedzi?

Wpisz...

Wstecz Dalej

Stwórz własną ankietę na Webankieta.pl

Wiedza, zachowania i postawy fizjoterapeutów wobec praktyki opartej na danych naukowych (EBP2Q)

38% — 100%

Jak często w ubiegłym roku: *

Można udzielić jednej odpowiedzi w wierszu.

	nigdy (1)	raz w miesiącu lub rzadziej (2)	raz na dwa tygodnie (3)	raz na tydzień (4)	codziennie (5)
39. Sformułowała Pani / sformułował Pan poprawne pytanie kliniczne dotyczące pacjenta, problemu, podjętych działań i ich wyników?					
40. Znalazła Pani / znalazł Pan dowody naukowe adekwatne do postawionego pytania?					
41. Korzystała Pani / korzystał Pan z elektronicznej bazy piśmiennictwa naukowego?					
42. Krytycznie oceniła Pani / ocenił Pan poprawność metodologiczną wykorzystanej literatury naukowej?					
43. Odniosła Pani / odniósł Pan wyniki badań naukowych do własnej diagnozy?					
44. Uwzględniała Pani / uwzględniał Pan preferencje pacjenta przy podejmowaniu decyzji klinicznych?					
45. Czytała Pani / czytał Pan wyniki opublikowanych badań naukowych?					
46. Nieformalnie omawiała Pani / omawiał Pan doniesienia naukowe ze współpracownikami?					
47. Formalnie omawiała Pani / omawiał Pan doniesienia naukowe podczas posiedzeń naukowych?					

Czy ma Pani / Pan dodatkowe komentarze dotyczące udzielonych odpowiedzi?

Wpisz...

Wstecz Dalej

Stwórz własną ankietę na Webankieta.pl

Wiedza, zachowania i postawy fizjoterapeutów wobec praktyki opartej na danych naukowych (EBP2Q)

46% — 100%

Proszę ocenić poziom umiejętności związanych z EBP: *

Można udzielić jednej odpowiedzi w wierszu.

	zdecydowanie nie umiem / nie potrafię (1)	nie umiem / nie potrafię (2)	nie mam zdania (3)	umiem, potrafię (4)	zdecydowanie umiem/ potrafię (5)
48. Umiejętności badawcze	○	○	○	○	○
49. Obsługa komputera	○	○	○	○	○
50. Zdolność do identyfikacji braków w wiedzy	○	○	○	○	○
51. Zdolność do formułowania poprawnych pytań klinicznych	○	○	○	○	○
52. Znajomość głównych typów informacji i ich źródeł	○	○	○	○	○
53. Umiejętność wyszukiwania informacji w elektronicznych bazach piśmiennictwa naukowego	○	○	○	○	○
54. Umiejętność uzyskania dostępu do dowodów naukowych (uzyskanie kopii artykułów lub raportów)	○	○	○	○	○
55. Umiejętność krytycznej analizy dowodów naukowych w świetle stosowanych norm	○	○	○	○	○
56. Umiejętność określenia stopnia wiarygodności uzyskanego dowodu naukowego	○	○	○	○	○
57. Zdolność do określenia stopnia użyteczności klinicznej uzyskanego dowodu naukowego	○	○	○	○	○
58. Umiejętność zastosowania informacji naukowej do poszczególnych przypadków (tj. zintegrowanie wyników badań z osobistymi preferencjami, wartościami, obawami i oczekiwaniami własnymi i pacjenta)	○	○	○	○	○

Czy ma Pani / Pan dodatkowe komentarze dotyczące udzielonych odpowiedzi?

Wpisz...

Wstecz Dalej

Stwórz własną ankietę na Webankieta.pl

Wiedza, zachowania i postawy fizjoterapeutów wobec praktyki opartej na danych naukowych (EBP2Q)

54% — 100%

Proszę ocenić trafność poszczególnych twierdzeń w skali od 1 do 5: *

Można udzielić jednej odpowiedzi w wierszu.

	zdecydowanie się nie zgadzam (1)	nie zgadzam się (2)	nie mam zdania (3)	zgadzam się (4)	zdecydowanie się zgadzam (5)
59. Chcę zdobywać nowe informacje	○	○	○	○	○
60. Krytycznie oceniam nowe pomysły	○	○	○	○	○
61. Mam predyspozycje do zarządzania	○	○	○	○	○
62. W rozwiązywaniu problemów posługuję się planem	○	○	○	○	○
63. Lubię się uczyć	○	○	○	○	○
64. W mojej pracy zawodowej kadra zarządzająca stale poszukuje nowych możliwości uczenia się	○	○	○	○	○
65. Znajduję czas na czytanie badań naukowych	○	○	○	○	○
66. Brak czasu jest jedną z największych barier uniemożliwiających wykorzystywanie EBP w mojej praktyce zawodowej	○	○	○	○	○
67. Obciążenie pracą zawodową uniemożliwia mi regularną aktualizację mojej wiedzy	○	○	○	○	○
68. Koszty użytkowania zasobów informacyjnych ograniczają zastosowanie EBP w praktyce zawodowej	○	○	○	○	○
69. Dostęp do komputera ma wpływ na zastosowanie EBP w mojej praktyce zawodowej	○	○	○	○	○
70. Wykorzystanie dostępnych źródeł wiedzy wystarcza do stosowania EBP w mojej praktyce zawodowej	○	○	○	○	○
71. Wsparcie współpracowników jest jedną z największych motywacji do stosowania EBP w praktyce zawodowej	○	○	○	○	○
72. Wsparcie ze strony kadry zarządzającej jest jedną z największych motywacji do stosowania EBP w praktyce zawodowej	○	○	○	○	○
73. Mój pracodawca wymaga ode mnie używania EBP w codziennej praktyce zawodowej	○	○	○	○	○
74. Mam już dosyć EBP	○	○	○	○	○

Czy ma Pani / Pan dodatkowe komentarze dotyczące udzielonych odpowiedzi?

Wpisz...

Wstecz | Dalej

Stwórz własną ankietę na Webankieta.pl

Ministerstwo Nauki i Szkolnictwa Wyższego
Krajowa Izba Fizjoterapeutów

Wiedza, zachowania i postawy fizjoterapeutów wobec praktyki opartej na danych naukowych (EBP2Q)

63% — 100%

Jestem *

Można udzielić jednej odpowiedzi.

- Kobietą
- Mężczyzną

Rok urodzenia *

Proszę podać Pani/Pana rok urodzenia.

Proszę podać liczbę całkowitą.

Wpisz...

W jakim aktualnie państwie Pani/Pan mieszka? *

Można udzielić jednej odpowiedzi.

- Polska
- Inne, jakie?

Województwo *

Proszę wskazać województwo, w którym Pani/Pan zamieszkuje.

Można udzielić jednej odpowiedzi.

- dolnośląskie
- kujawsko-pomorskie
- lubelskie
- lubuskie
- łódzkie
- małopolskie
- mazowieckie
- opolskie
- podkarpackie
- podlaskie
- pomorskie
- śląskie
- świętokrzyskie
- warmińsko-mazurskie
- wielkopolskie
- zachodniopomorskie
- nie dotyczy (mieszkam poza Polską)

Poziom wykształcenia *

Proszę wybrać uzyskany poziom wykształcenia zgodnie z zapisami ustawy o zawodzie fizjoterapeuty.

Można udzielić jednej odpowiedzi.

- technik fizjoterapii
- licencjat fizjoterapii
- magister fizjoterapii
- magister fizjoterapii ze specjalizacją

Stopień/tytuł naukowy (jeśli dotyczy) *

Proszę zaznaczyć najwyższy posiadany.

Można udzielić jednej odpowiedzi.

- dr
- dr hab.
- prof.
- nie dotyczy

Proszę podać staż pracy w zawodzie fizjoterapeuty. *

Można udzielić jednej odpowiedzi.

- do 1 roku
- od 1 do 3 lat
- od 3 do 5 lat
- od 6 do 10 lat
- od 11 do 19 lat
- 20 lat lub więcej
- Aktualnie nie pracuję w zawodzie fizjoterapeuty

Gdzie głównie Pani/Pan pracuje? *

Można zaznaczyć kilka odpowiedzi.

- [ ] Szpital, klinika
- [ ] Przychodnia, zakład rehabilitacji
- [ ] Rehabilitacja domowa
- [ ] ZOL, DPS
- [ ] Prywatny gabinet
- [ ] Sanatorium
- [ ] Uczelnia
- [ ] Klub sportowy, siłownia
- [ ] Inne miejsce, jakie?

Wstecz | Dalej

Stwórz własną ankietę na Webankieta.pl

Wiedza, zachowania i postawy fizjoterapeutów wobec praktyki opartej na danych naukowych (EBP2Q)

96% — 100%

Miejscowość *

Jaka jest wielkość miejscowości, w której wykonuje Pani/Pan usługi fizjoterapeutyczne (w przypadku pracy w różnych typach miejscowości proszę podać dla głównego miejsca zatrudnienia).

Można udzielić jednej odpowiedzi.

- ○ Miasto pow. 500 tys. mieszkańców
- ○ Miasto 100 - 499 tys. mieszkańców
- ○ Miasto 20 - 99 tys. mieszkańców
- ○ Miasto do 20 tys. mieszkańców
- ○ Wieś

Wstecz | Wyślij

Stwórz własną ankietę na Webankieta.pl

Dziękujemy za wypełnienie ankiety

Stwórz własną ankietę na Webankieta.pl
