# Supplementary material for: Knowledge, behaviours and attitudes towards Evidence-Based Practice amongst physiotherapists in Poland. A nationwide cross-sectional survey and focus group study protocol
Source: PLoS One. 2022 Mar 1;17(3):e0264531. doi: 10.1371/journal.pone.0264531 (PMC8887773; doi:10.1371/journal.pone.0264531)
Supplement: S3 File — (PDF) [file pone.0264531.s003.pdf]

Template table to be used for Study 2 – focus group discussions.

Themes with illustrative quotes:

Theme	Illustrative quotation
**Understanding of EBP**	
**Opinions on EBP**	
**Using EBP in the workplace**	
**EBP in daily practice**	
