# Supplementary material for: Knowledge, behaviours and attitudes towards Evidence-Based Practice amongst physiotherapists in Poland. A nationwide cross-sectional survey and focus group study protocol
Source: PLoS One. 2022 Mar 1;17(3):e0264531. doi: 10.1371/journal.pone.0264531 (PMC8887773; doi:10.1371/journal.pone.0264531)
Supplement: Attachment — Submitted filename: review of PLOS ONE manuscript.docx [file pone.0264531.s004.docx]

**Manuscript Number: PONE-D-21-25941**

**Title:** **Knowledge, behaviours and attitudes towards Evidence-Based Practice amongst physiotherapists in Poland. A nationwide cross-sectional survey and focus group study protocol**

**Comment 1**: this is research proposal why don’t you consider its publication after study was conducted?

**Comment 2**: on page 2 at the end of first paragraph of your abstract you have mentioned as this title/issue was addressed in other parts of the world. So why don’t you use already existed evidences or why not consider doing systematic review and meta- analysis from these evidences.

**Comment 3:** on page 2 under methods of abstract you have planned to use online survey. One big problem of this method was respondent may update him/her self before responding and may discuss with others. So how you will overcome these problems? You also mentioned use of purposive sampling. Did you think purposive sampling can help in assuring representativeness? Why don’t you consider sampling methods like stratified sampling techniques?

Comment 4: on page 3 just above objectives you have said there is no same study in Poland. Why did you take this as justification to repeat the study? Is there any difference you have found between Poland and other countries physiotherapists?. Add more strong justification to repeat the study.

Comment5: on page 5 under sample size you just plan to take 1000 participants. but you better show clear procedure on how you calculate your sample size. So it help future researcher in replication of the study.

Comment 6: on page 6 under Data management and analysis you have planned to run descriptive statistics. But you should specify what types of descriptive statistics for groups of variables (Example for continuous data and for categorical data).

Comment 7: on page 8 under discussion you wrote a lot of evidence regarding evidence based practice. Did you think you will find different results despite you didn’t mention weakness of previous studies.

Comment 8: on page 9 under limitation you put anticipated limitation. But in reality what you think may not come problematic. Please consider writing limitation after completion of the study.

Comment: 9 on page 10 under author contribution you write some contribution which will expect if study was already conducted (e.g Formal analysis: Maciej Płaszewski, Weronika Grantham, Joanna Trębska). Please revise.

Reviewer: Ahmednur Adem Aliyi

Email: [ahmedhariro@gmail.com](mailto:ahmedhariro@gmail.com)
