# Supplementary material for: Knowledge, behaviours and attitudes towards Evidence-Based Practice amongst physiotherapists in Poland. A nationwide cross-sectional survey and focus group study protocol
Source: PLoS One. 2022 Mar 1;17(3):e0264531. doi: 10.1371/journal.pone.0264531 (PMC8887773; doi:10.1371/journal.pone.0264531)
Supplement: Attachment — Submitted filename: PLOS ONE paper review 4.docx [file pone.0264531.s005.docx]

**PONE-D-21-25941**

**Re: “Knowledge, behaviors and attitudes towards Evidence-Based Practice amongst physiotherapists in Poland. A nationwide cross-sectional survey and focus group study protocol”**

This research protocol is well designed outlying the planning of the authors’ study. I have the following **Comments:**

**Abstract:** conclusions should be rewritten to explain the proposed or expected outcome rather than repeating the purpose and objectives of the study under conclusions.

**Introduction:** should be rewritten. The authors quoted 40 references under introduction with only four short paragraphs. Start with the definition of EBP and the introduction will benefit from detailed explanation.

The authors mentioned at the end of introduction “In contrast, no such research was completed in Poland. This is the first study to address EBP profiles of physiotherapists practicing in Poland”. This is a strong statement and cannot be granted as the authors unaware of unpublished work in this field. I suggest deleting this statement.

**Objectives:** under specific objectives please add “ To formulate recommendations based on the findings of the study”

**Methods:**

**Quantitative study:** The validity of questionnaire should be obtained from reference 42 and their Cronbach’s alpha and reliability scores should be reported after data collection.

Explain in more details’ inclusion and exclusion criteria of the participants.

What is the proposed or estimated response rate among the participants?

Under setting and participants, second paragraph: the statement “ A small number of physiotherapists ……” How many? Need reference to support it.

Table 1: outline the presence of many confounding factors with focus on work experience and highest qualifications which are related to outcome of KAP.

Under sample size: How the data will be extracted? What will be the sampling technique? What is the sampling equation to calculate sample size? How did the authors come up with around 1000?

Under data collection: what are the dependent and independents variables?

I have a concern with 5-point Likert scale as it has too many available categories which may obscure rather than define the purpose of the respondent. Therefore, I suggest collapsing into 3 categories across response which may improve the outcome of the analysis (Reference: Grimbeek P., Bryer F., Beamish W., D’Netto M. Stimulating the’Action’ as Participants in Participatory Research. Volume 2. Griffith University; Brisbane, Australia: 2005. Use of data collapsing strategies to identify latent variables in CHP questionnaire data; p. 125.).

Furthermore, each response in the scale should be assigned a numerical value so that parametric statistics can be used for Likert data. Higher score indicates positive response.

**Qualitative study**: this will definitely complement the quantitative study; however, it may be a problematic during COVID-19 pandemic. If this is the case, those conducting the interview should implement physical distance restrictions and wear face mask during the interview to overcome this problem.

What is the duration of the interview? When will it take place? Over span of how long?

The one conducting interview should have experience in qualitative research

Experts in qualitative research should supervised data collection and hold feedback sessions shortly after the interview

All interviews should be recorded and transcribed in full to verify the accuracy of responses

Two members of the research team independently should analyze the transcribed responses and read them on multiple times to familiarize self with the contents and categorize it in a meaningful way.

All these points should be explained and incorporated under the design of qualitative study.

**Limitations of the study:** should explain in details and logical ways. Personal, recall, misinterpretation biases are possibilities in this study

**References:** The manuscript presents several references which are outdated (1 and 44), and some are irrelevant. The references should be revised, updated, and only used if it is relevant to the study. It also should follow the guidelines of the journal.
