# Supplementary material for: Knowledge, behaviours and attitudes towards Evidence-Based Practice amongst physiotherapists in Poland. A nationwide cross-sectional survey and focus group study protocol
Source: PLoS One. 2022 Mar 1;17(3):e0264531. doi: 10.1371/journal.pone.0264531 (PMC8887773; doi:10.1371/journal.pone.0264531)
Supplement: Attachment — Submitted filename: re 2 for EBP.docx [file pone.0264531.s007.docx]

**Title:** Knowledge, behaviours and attitudes towards Evidence-Based Practice amongst physiotherapists in Poland.  A nationwide cross-sectional survey and focus group study protocol.

**Dear author of this study,**

I will not pass every effort you made in responding to comments and correction you made in your documents.

But I believe you should modify your last objective which say “to identify the facilitators and barriers in the implementation of EBP they experience in their everyday practice” . Because you planned to use crossectional study (survey) which can’t tell us direction of relationships. I recommend you to modify like “ to identify factors associated with EBP among physiotherapist”
