# Supplementary material for: Knowledge, behaviours and attitudes towards Evidence-Based Practice amongst physiotherapists in Poland. A nationwide cross-sectional survey and focus group study protocol
Source: PLoS One. 2022 Mar 1;17(3):e0264531. doi: 10.1371/journal.pone.0264531 (PMC8887773; doi:10.1371/journal.pone.0264531)
Supplement: Attachment — Submitted filename: Response to Reviewers.docx [file pone.0264531.s008.docx]

**Manuscript Number: PONE-D-21-25941**

***Title: Knowledge, behaviours and attitudes towards Evidence-Based Practice amongst physiotherapists in Poland. A nationwide cross-sectional survey and focus group study protocol***

Dear Academic Editor,

Dear Reviewers,

We would like to thank you for the analysis of our revised manuscript and for your valuable feedback. Please find below our responses to your comments.

**Response to the Reviewer #1:**

***Reviewer’s comment:***

*The authors have done a good job responding to the reviewers and editing the study protocol accordingly. I only have a few final suggestions.*

*While the author’s have partially addressed the motivation behind the study beyond that it is the first of its kind in Poland, I still think the manuscript could benefit from a little bit stronger motivation. For example, on page 3 in the statement “to the best of our knowledge, no such research was completed in Poland” could be enhanced with something like “and there are unique factors of this context which may be important” etc. Also, the abstract still lists the study being in Poland as the only real motivation.*

**Our response:**

We thank the Reviewer for this suggestion. We added a more detailed justification to the sentence you indicated. We also added an explanatory statement in the abstract, in the ‘Objectives’ section.

***Reviewer’s comment:***

*There are still some grammatical mistakes and sentences which could be edited.*

**Our response:**

The manuscript was double-checked by our native English colleague. He still found some inaccuracies and they are now rewritten. Thank you for this remark.

***Reviewer’s comment:***

*The authors bring up the Hawthorne effect in the limitations section. It could be useful to the reader to briefly explain what this is (that is, that people change their behavior or answer differently when being observed).*

**Our response:**

Thank you for this suggestion. We have added a short explanation, following the Hawthorne effect mention.

***Reviewer’s comment:***

*Finally, the abstract now only consists of objectives and methods, I understand this because it is a protocol, but would it be possible to just have an unstructured abstract instead?*

**Our response:**

Thank you for this suggestion, but we would like to leave the structured layout of the protocol, as standard fot PLoS ONE, including registered report protocols, such as Spitzer L, Mueller S (2021) Registered Report Protocol: Survey on attitudes and experiences regarding preregistration in psychological research. PLoS ONE 16(7):e0253950 and Adams D, Malone S, Simpson K, Tucker M, Rapee RM, Rodgers J, et al. (2021) Protocol for a longitudinal study investigating the role of anxiety on academic outcomes in children on the autism spectrum. PLoS ONE 16(9): e0257223, which also have no ‘Conclusions’ sections.

**Response to the Reviewer #2:**

***Reviewer’s comment:***

*Dear author of this study,*

*I will not pass every effort you made in responding to comments and correction you made in your documents.*

*But I believe you should modify your last objective which say “to identify the facilitators and barriers in the implementation of EBP they experience in their everyday practice” . Because you planned to use crossectional study (survey) which can’t tell us direction of relationships. I recommend you to modify like “ to identify factors associated with EBP among physiotherapist”*

**Our response:**

We thank the Reviewer for this remark. We have rewritten the objective so that it is now not suggesting an explanatory, rather than a descriptive study.

We would like to thank the Reviewer 3 and the Reviewer 4 for analysing our revised manuscript and for their replies.

We are grateful to the Reviewers and to the Academic Editor for their valuable input to our manuscript. Once again, we do appreciate your expert support. Our paper, and our study, have considerably improved through this peer-review process.
