# Supplementary material for: Knowledge, behaviours and attitudes towards Evidence-Based Practice amongst physiotherapists in Poland. A nationwide cross-sectional survey and focus group study protocol
Source: PLoS One. 2022 Mar 1;17(3):e0264531. doi: 10.1371/journal.pone.0264531 (PMC8887773; doi:10.1371/journal.pone.0264531)
Supplement: Attachment — Submitted filename: Response to Reviewers.docx [file pone.0264531.s009.docx]

Dear Reviewers,

Thank you for your final analysis of our manuscript. We are thankful for your valuable input to this paper and this project. Thank you.

Dear Academic Editor,

Thank you for the points you have raised to our revised manuscript. And thank you for the time and effort you have given to the whole process of scientific editing of our submission.

Please find below our elaboration on how we addressed your suggestions in the amended manuscript.

***Editor comment:***

*Editing the abstract: deleting duplications of methods in the online version, and adding something on the results (i.e. how they will be presented) and discussion (i.e. limitations).*

**Our response:**

Based on your suggestions, we have made the following changes:

- Both the online, and paper, versions, are now identical.
- The Abstract, especially the issue of whether to include Results and Discussion/Conclusions sections in the Abstract had been previously raised by two of the Reviewers and we have already changed the abstract, especially the sections under discussion. Therefore, while preparing this revision, we took those comments and suggestions of the Reviewers into consideration. Nonetheless, we think that your suggestion is final and decisive in this matter, especially in terms of the Journal’s requirements, so we have added the Results and Discussion as you have indicated.

The word count for Abstract is 300 in the revised manuscript.

***Editor comment:***

*Making presentation plan consistent for the survey and the qualitative study (i.e. objective, methods, results); please note, I believe that it’s easier for the reader to see the plan for the analysis and results presentation (e.g. empty tables) separately, as it would be in a research article.*

**Our response:**

Thank you for this suggestion. We have analysed the paper again in the context of the separate presentation of the two legs of our study (the survey, Study 1, and the focus group study, Study 2) and we have decided as follows:

- we believe the layout of the paper, as it is already provided, is clear and understandable in terms of the separate description of the objectives and methods of Study 1 and Study 2: we provide separate sections (i.e. *QUANTITATIVE STUDY (Study 1); QUALITATIVE STUDY (Study 2)*) and subsections to both sections (such as Objectives, Methods, Study design, Data collection and analysis); but, we are aware that reporting two planned studies in one paper is challenging and we do understand your concern, so we have decided to add to the manuscript a supplementary file – the flow chart of the study, with a graphic presentation of the planned study conduct, with its quantitative and qualitative part; we have added a corresponding statement in the paper, at the beginning of the Methods section, before the description of Study 1 and Study 2; we believe it will serve as an additional guide for the readers;
- as regards the results and planned analyses, we have provided an additional table – also as a supplementary file – a template table which will be used during the focus group meetings for data (narratives) collection and then for analyses of the qualitative part (Study 2); we have added (in the Data collection section) a relevant statement in the paper – leading to the supplementary file (Supplementary File 3);
- as regards the survey part (Study 1) the manuscript includes a demographics table and an EBP^2^Q structure and content table, which, when used with the template survey (supplementary file – already included, now Supplementary File 2), provides in our view a sufficient guide for the readers of how the data will be collected and then how the results will be presented.

Again, thank you very much for your support and guidance. We believe that the provided improvements will meet the requirements of the Journal. For a technical tip from the Journal, please see below our reply.

***Journal requirements:***

*Please review your reference list to ensure that it is complete and correct. If you have cited papers that have been retracted, please include the rationale for doing so in the manuscript text, or remove these references and replace them with relevant current references. Any changes to the reference list should be mentioned in the rebuttal letter that accompanies your revised manuscript. If you need to cite a retracted article, indicate the article’s retracted status in the References list and also include a citation and full reference for the retraction notice.*

**Our response:**

We have revised our reference list. It is complete and correct. There are no citations to retracted papers. We have not made any changes to the reference list.
